# Interpersonal Psychotherapy (IPT) for Eating Disorders: A Literature Review

**DOI:** 10.1192/j.eurpsy.2025.2136

**Published:** 2025-08-26

**Authors:** M. L. Aires, J. Barreira, D. Stolnik, M. Pinto

**Affiliations:** 1 Unidade Local de Saúde Tâmega e Sousa, Porto, Portugal

## Abstract

**Introduction:**

Interpersonal Psychotherapy (IPT) has been recognized as an effective treatment for various eating disorders, particularly Binge Eating Disorder (BED) and Bulimia Nervosa (BN). IPT focuses on addressing interpersonal difficulties, such as role transitions, interpersonal disputes, and social deficits, which are common in individuals with eating disorders. While evidence supports its use for BED and BN, its effectiveness for Anorexia Nervosa (AN) remains limited. Group-based IPT has also shown promise by targeting interpersonal deficits that perpetuate disordered eating behaviors.

**Objectives:**

This literature review aims to evaluate the efficacy of IPT in treating eating disorders, with a focus on BED and BN. Additionally, it explores the role of interpersonal deficits as a central focus of IPT and compares the outcomes of individual and group-based IPT formats.

**Methods:**

A review of peer-reviewed studies, clinical trials, and meta-analyses published between 2000 and 2024 was conducted. The review assessed the effectiveness of IPT in reducing disordered eating behaviors, improving interpersonal functioning, and maintaining long-term treatment gains. Studies on both individual and group IPT formats were included, with a focus on binge eating reduction, interpersonal relationship improvements, and relapse prevention.

**Results:**

The literature consistently shows that IPT is effective in reducing binge eating behaviors and improving interpersonal relationships in individuals with BED. Group IPT, in particular, has been shown to effectively address interpersonal deficits, providing social support and improving interpersonal skills, which contributes to sustained treatment outcomes. IPT has demonstrated comparable short-term efficacy to Cognitive Behavioral Therapy (CBT), with studies indicating superior long-term maintenance of treatment effects. However, evidence for IPT’s use in AN remains sparse.

**Conclusions:**

IPT is a promising treatment for eating disorders, particularly for individuals with BED and BN who exhibit significant interpersonal deficits. Group IPT appears to be especially effective in addressing these interpersonal issues, providing long-term benefits. While CBT remains the most widely used therapy, IPT offers a valuable alternative, particularly for individuals who do not respond to CBT. Future research should focus on exploring IPT’s mechanisms and expanding its application to other eating disorders, including AN, where evidence is currently lacking.

**Disclosure of Interest:**

None Declared

